# Elucidation of Interaction between Whey Proteins and Proanthocyanidins and Its Protective Effects on Proanthocyanidins during In-Vitro Digestion and Storage

**DOI:** 10.3390/molecules26185468

**Published:** 2021-09-08

**Authors:** Chenyu Tang, Bing Tan, Xiangjun Sun

**Affiliations:** Department of Food Science and Engineering, School of Agriculture and Biology, Shanghai Jiaotong University, Shanghai 200240, China; GloriaTang@sjtu.edu.cn (C.T.); tanbing001@sjtu.edu.cn (B.T.)

**Keywords:** proanthocyanidins, whey protein, interaction, spectroscopy, molecular docking, stability

## Abstract

Whey proteins and oligomeric proanthocyanidins have nutritional value and are widely used in combination as food supplements. However, the effect of the interactions between proanthocyanidins and whey proteins on their stability has not been studied in depth. In this work, we aimed to characterize the interactions between β-Lactoglobulin (β-LG) and α-lactalbumin (α-LA) and oligomeric proanthocyanidins, including A1, A2, B1, B2, B3, and C1, using multi-spectroscopic and molecular docking methods. Fluorescence spectroscopic data revealed that all of the oligomeric proanthocyanidins quenched the intrinsic fluorescence of β-LG or α-LA by binding-related fluorescence quenching. Among the six oligomeric proanthocyanidins, A1 showed the strongest affinity for β-LG (K_a_ = 2.951 (±0.447) × 10^4^ L∙mol^−1^) and α-LA (K_a_ = 1.472 (±0.236) × 10^5^ L∙mol^−1^) at 297 K. β-LG/α-LA and proanthocyanidins can spontaneously form complexes, which are mainly induced by hydrophobic interactions, hydrogen bonds, and van der Waals forces. Fourier-transform infrared spectroscopy (FTIR) and circular dichroism spectroscopy showed that the secondary structures of the proteins were rearranged after binding to oligomeric proanthocyanidins. During in vitro gastrointestinal digestion, the recovery rate of A1 and A2 increased with the addition of WPI by 11.90% and 38.43%, respectively. The addition of WPI (molar ratio of 1:1) increased the retention rate of proanthocyanidins A1, A2, B1, B2, B3, and C1 during storage at room temperature by 14.01%, 23.14%, 30.09%, 62.67%, 47.92%, and 60.56%, respectively. These results are helpful for the promotion of protein–proanthocyanidin complexes as functional food ingredients in the food industry.

## 1. Introduction

Proanthocyanidins are condensed polyphenol polymers of monomeric flavan-3-ols, and they are widely found in fruits, berries, nuts, and seeds [1]. Proanthocyanidins are effective against cancer, inflammation, cardiovascular disease, type 2 diabetes, and autoimmune diseases because of their powerful antioxidant activities, as well as their targeted protein-binding and cell signaling pathway-regulating abilities [2,3,4]. Among the kinds of proanthocyanidins, dimer proanthocyanidins linked via C4-C8 or C4-C6 bonds (B-type) or via additional C2-O-C7 or C2-O-C5 bonds (A-type) and trimer proanthocyanidin C1 have received considerable attention because of their abundance in plants. Additionally, these proanthocyanidins, with a degree of polymerization of no more than three, have great potential as functional foods. They may penetrate the intestinal wall and can be detected in human plasma [5]. The majority of ingested proanthocyanidins can reach the colon and be degraded by gut microflora, and the microbial metabolites of proanthocyanidins have a number of health benefits [6,7]. However, proanthocyanidins are unstable in the alkaline environment of intestine [8], which limits their healthful function in vivo. Moreover, food proanthocyanidins can be damaged by high temperature, oxygen, and irradiation during processing or storage [7]. Therefore, maintaining the stability of proanthocyanidins during processing, storage, and gastrointestinal digestion to maximize their nutritional value is of great importance.

Exploiting chemical interactions with proteins has proven to be an effective strategy for improving the stability of food polyphenols [9]. Among these proteins, whey protein has widespread applications in the food industry as a texture modifier, emulsifier, and gelling agent [10]. Moreover, it is added to infant formulas, sports foods, and functional foods because of its high bioavailability and considerable relative abundance of essential and non-essential amino acids [11]. β-Lactoglobulin (β-LG) and α-lactalbumin (α-LA) are the main whey protein components, constituting 65% and 25%, respectively. Whey proteins and oligomeric proanthocyanidins collectively improve the nutritional value of food if added in combination. Moreover, whey proteins can be used as delivery vehicles for oligomeric proanthocyanidins. Several studies investigated the interactions between proanthocyanidins and whey proteins. Wang et al. [12] reported that the interaction between procyanidin B2 and peptides of α-lactalbumin prevented procyanidin B2 from degradation. Hu et al. [13] demonstrated that the superior antioxidant activity of proanthocyanidins in the WPI-stabilized oil system could be due to their ability to bind to whey protein. Chen et al. [14] showed that lotus seedpod proanthocyanidin–whey protein complexes can be used as effective emulsifiers and antioxidants, which may be useful for developing more efficacious functional foods and beverages. However, the mechanism of the interactions between proanthocyanidins and whey protein or its main components, including β-LG and α-LA, has not been elucidated. Additionally, the specific effect of individual proanthocyanidin configuration on the affinity towards proteins is unclear.

This study aimed to characterize the interactions between β-LG or α-LA and six types of oligomeric proanthocyanidins (B1, B2, B3, A1, A2, and C1, Figure S1) by using multi-spectroscopic and molecular docking methods. Further exploration of the interactive effects of whey proteins on the stability of the proanthocyanidin during gastrointestinal digestion and storage were carried out, and the findings might be useful in the development of functional foods.

## 2. Results and Discussion

### 2.1. Analysis of Fluorescence Quenching

Tryptophan (Trp), tyrosine (Tyr), and phenylalanine (Phe) residues are intrinsic fluorescent groups of protein, and they are sensitive to microenvironmental changes. When a protein interacts with a quencher, the fluorescence efficiency or lifetime of the excited state may decrease. Thus, the fluorescence intensity decreases.

#### 2.1.1. Fluorescence Spectra of WPI Bound to Proanthocyanidins

**Figure 1:**
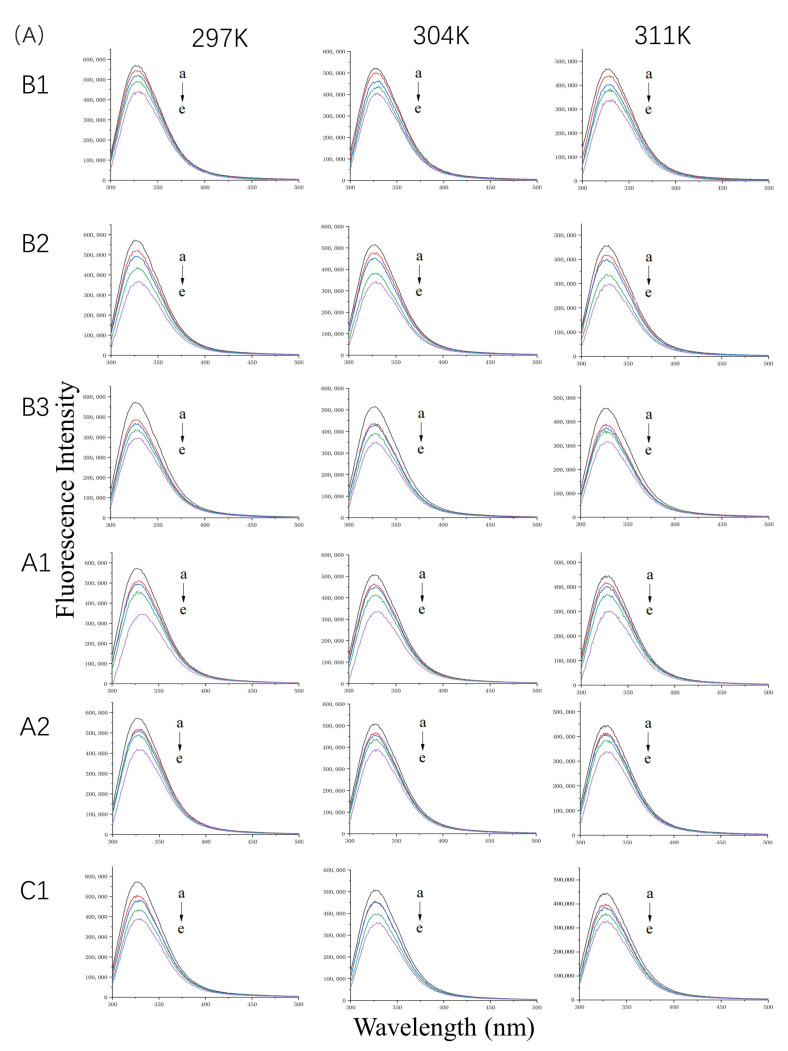

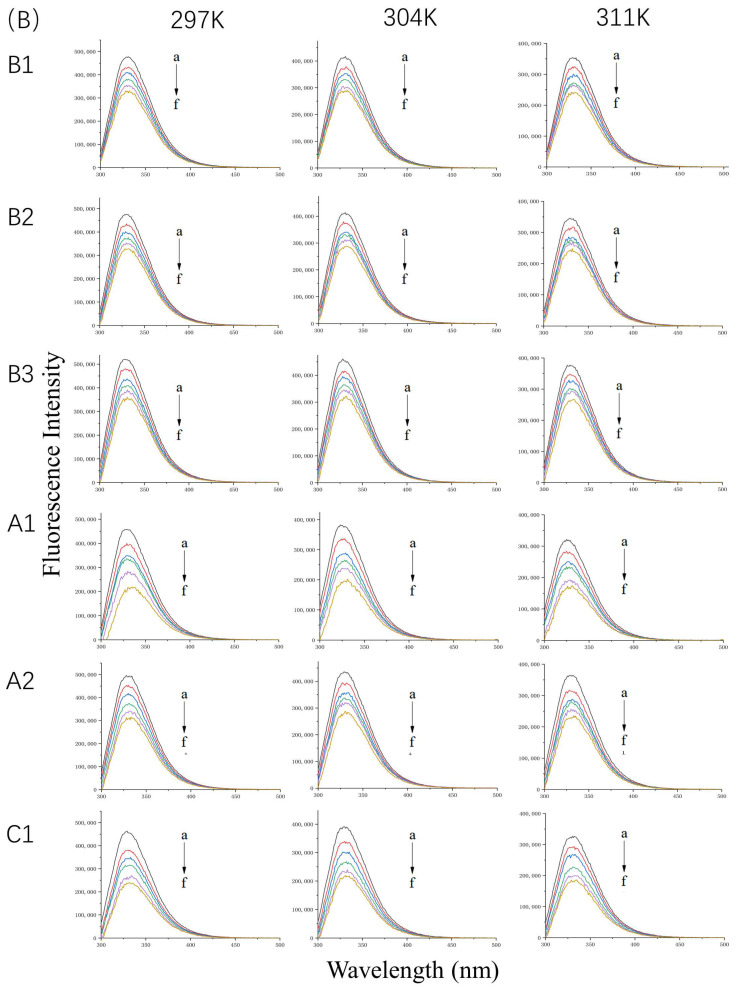

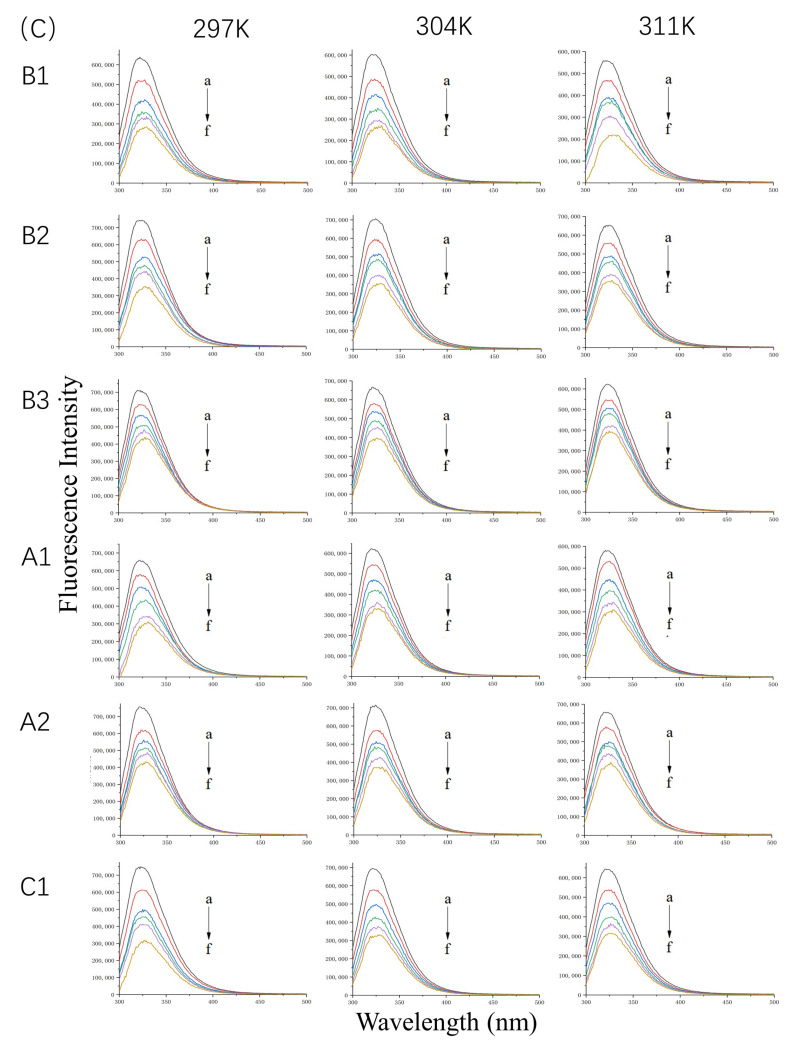
Fluorescence spectra of 10 µM WP (**A**) in the presence of 0, 5, 10, 20, and 40 µM (a–e) proanthocyanidins A1, A2, B1, B2, B3, and C1 and 20 µM β-LG (**B**) or α-LA (**C**) in the presence of 0, 10, 20, 30, 40, and 50 µM (a–f) proanthocyanidins A1, A2, B1, B2, B3, and C1 at 297 K, 304 K, and 311 K and pH 6.3.

The fluorescence emission spectra of WPI at 297 K, 304 K, and 311 K in the absence or presence of proanthocyanidins are shown in Figure 1A. At a constant temperature, the quenching effect was enhanced with an increase in proanthocyanidin concentration in the solution. The maximum quenching rate of A1, A2, B1, B2, B3, and C1 was 39.66%, 26.72%, 35.90%, 31.85%, 27.40%, and 31.45%, respectively. The fluorescence spectra showed that interactions exist between WPI and proanthocyanidins. However, Stern–Volmer plots were not linear (Figure S2) because of the diversity of proteins in WPI, indicating mixed quenching mechanisms. In order to accurately and deeply explore the interactions between whey proteins and proanthocyanidins, β-LG and α-LA, the two main components of WPI were selected for the next experiments.

#### 2.1.2. Fluorescence Spectra of β-LG or α-LA Bound to Proanthocyanidins

The fluorescence emission spectra of β-LG and α-LA at 297 K, 304 K, and 311 K in the absence or presence of proanthocyanidins are shown in Figure 1B,C. The maximum quenching rate was observed at a molar ratio (i.e., protein-to-proanthocyanidin ratio) of 1:5 at 297 K. For β-LG, the quenching rate of A1, A2, B1, B2, B3, and C1 was 47.5%, 36.24%, 31.16%, 30.30%, 32.73%, and 49.67%, respectively. For α-LA, the quenching rate of A1, A2, B1, B2, B3, and C1 was 53.07%, 53.34%, 55.33%, 52.26%, 39.82%, and 55.32%, respectively, which is higher than that for β-LG. The data illustrate strong interactions between β-LG/α-LA and the six proanthocyanidins. Moreover, the proanthocyanidins quenched more fluorophores in α-LA than in β-LG. As the concentration of proanthocyanidins increased, the λ_max_ of β-LG showed a slight red shift of about 2 nm. The shift may have been associated with the microenvironmental changes of Trp19, located in the hydrophobic inner cavity, contributing to 80% fluorescence intensity [15]. In contrast, the λ_max_ of α-LA shifted from 322 nm to about 328 nm, which indicates that the microenvironment near Trp residues of α-LA became more hydrophilic [16].

**Figure 2:**
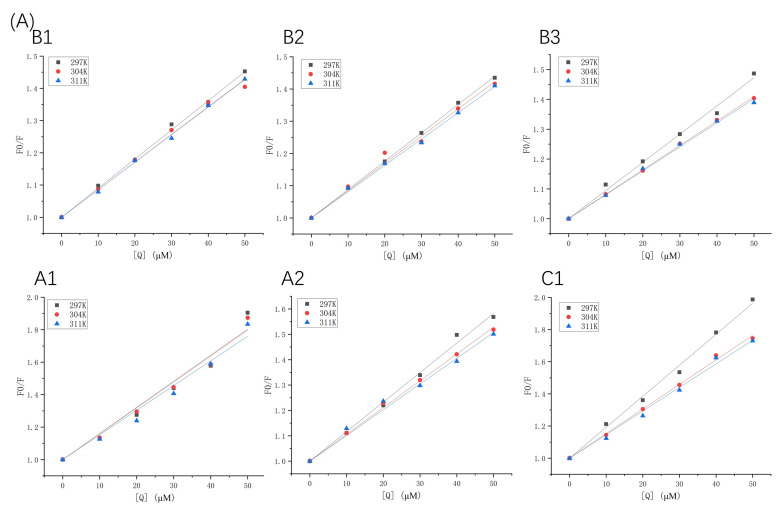

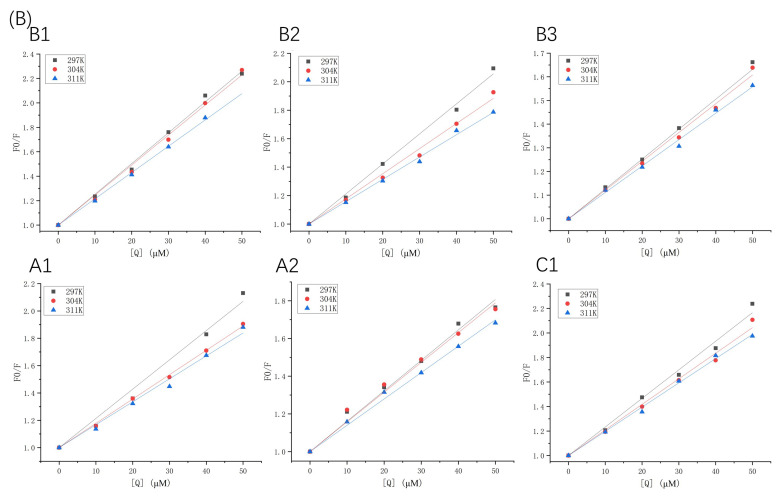
Stern–Volmer plots of β-LG (**A**) or α-LA (**B**) quenched by proanthocyanidins A1, A2, B1, B2, B3, and C1 at 297 K, 304 K, and 311 K.

Fluorescence quenching types can be described using the Stern–Volmer equation (Equation (1)). The Stern–Volmer plots of β-LG and α-LA, quenched by various concentrations of proanthocyanidins at different temperatures (297 K, 304 K, and 311 K), are displayed in Figure 2, and the quenching parameters are listed in Table 1 and Table 2. Fluorescence quenching can be classified into two types: the quenching caused by diffusion and collision between molecules, and binding-related quenching. The K_q_ values of proanthocyanidins surpassed one or two orders of magnitude of the maximum diffusion collision quenching constant (2 × 10^10^ M^−1^ s^−1^), which excludes the mechanism of collisional quenching [17]. Moreover, K_sv_ was inversely proportional to the temperature changes, which is also a criterion for validating the binding-related quenching mechanism [18]. In contrast, the collisional quenching mechanism has the opposite trend [19].

**Figure 3:**
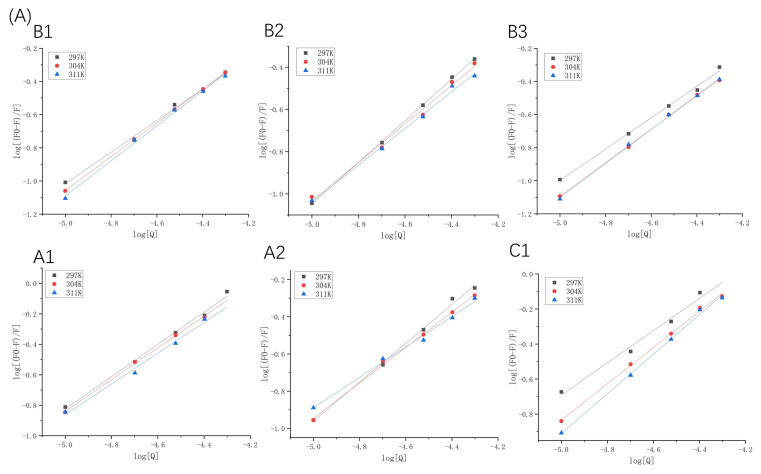

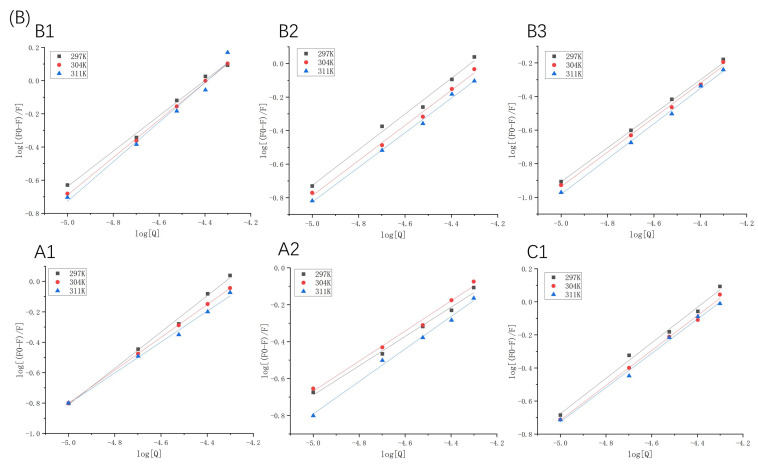
Double logarithmic Stern–Volmer plots of β-LG (**A**) or α-LA (**B**) quenched by proanthocyanidins A1, A2, B1, B2, B3, and C1 at 297 K, 304 K, and 311 K.

The fluorescence excitation was carried out at 280 nm; however, proanthocyanidins may also have an absorption at this wavelength. This indicates that an inner filter effect (IFE) may occur [20]. In order to evaluate whether IFE had a strong effect on the fluorescence quenching, the UV-vis absorption spectra of proanthocyanidin A1, A2, B1, B2, B3, C1, β-LG, and α-LA was detected in the range of 250–300 nm (Figure S3). The UV absorption of proanthocyanidins is much lower than that of β-LG and α-LA at 280 nm. It is worth mentioning that the absorption of proanthocyanidin C1 is higher than the other five proanthocyanidins. Generally, IFE causes a linear deviation of the Stern–Volmer equation and the value of K_a_ calculated according to fluorescence quenching will be larger than the actual one. However, our results showed that the R^2^ values of the Stern–Volmer equation in logarithmic form were above 0.99 in the range of the concentration of the quenchers (Figure 3). Moreover, compared with other five proanthocyanidins, the K_a_ value of proanthocyanidin C1 with proteins was relatively low (Table 1 and Table 2). The results show that the absorbance of proanthocyanidins at 280 nm did not have a significant effect on fluorescence quenching. Taken together, the fluorescence quenching was caused by the binding of proanthocyanidins to proteins and not by the inner-filtering effect.

#### 2.1.3. Binding Ability of β-LG or α-LA to Proanthocyanidins

In binding-related quenching, thermodynamic equilibrium is eventually reached between free and bound molecules. The double logarithm regression curves based on Equation (2) are shown in Figure 3, and the K_a_ and n values are presented in Table 1 and Table 2. The K_a_ values manifested different trends, which were attributed to dominant non-covalent forces between the two proteins and proanthocyanidins. For β-LG at 297 K, the K_a_ values of proanthocyanidins were arranged as A1 > A2 > C1 > B2 > B1 > B3, indicating that the binding forces between β-LG and A-type proanthocyanidins were significantly stronger than those between β-LG and B- and C-type proanthocyanidins. For α-LA at 297 K, the K_a_ values of proanthocyanidins were arranged as A1 > B1 > C1 > B2 > B3 > A2. The K_a_ of α-LA with proanthocyanidins was higher than that of β-LG (except A2) with proanthocyanidins. At pH 6.3, α-LA (pI = 4.2) carried more negative charges than β-LG (pI = 5.2), whereas the proanthocyanidins were weakly acidic [21]. This indicates that electrostatic interactions may have partly resulted in stronger affinity of proanthocyanidins toward α-LA.

Moreover, the interaction between A1 and two proteins was stronger than that between the other five oligomeric proanthocyanidins and two proteins. This is inconsistent with our previous findings, in which the interaction between A1 and β-casein was not detected [22]. The result indicates that the binding affinity of A-type proanthocyanidins to different milk proteins varies. Although the additional ether bond in A-type proanthocyanidins seemed to constrain the flexibility of the molecule, it simultaneously exposed the two rotatable catechol rings [23], which may have had a positive effect on the binding affinity. Inconsistencies in the binding ability of A2 with the two proteins may have been related to binding forces [24].

No significant difference was observed in the binding ability of B-type (dimer) or C-type (trimer) proanthocyanidins with the proteins (Table 1 and Table 2). This is inconsistent with a previous study, in which the molecular weight and number of hydroxyl groups of polyphenols were important in protein–polyphenol interactions [25]. The following can be an explanation for this: The higher degree of polymerization of proanthocyanidins provided multiple active sites for the interaction with proteins but also resulted in steric hindrance. The highly polymerized proanthocyanidins (molecular weight > 3400 Da) showed fewer effective interactions with the proteins [26].

In general, the characteristics of proteins, including hydrophobicity, isoelectric point, amino acid sequence, and molar flexibility, affect their binding affinity to oligomeric proanthocyanidins. However, the hydrophobicity and conformation of proanthocyanidins affect the formation of protein–polyphenol complexes.

#### 2.1.4. Thermodynamic Parameters and Binding Forces between Proteins and Proanthocyanidins

The ΔH, ΔS, and ΔG values were calculated using Equation (3) and Equation (4). The negative ΔG values proved that the formation of the protein–proanthocyanidin complex was spontaneous (Table 1 and Table 2). The ΔG value relates to the spontaneity of the complex formation and corresponds to the strength of the binding ability. Additionally, the binding forces between protein and quencher can be determined by thermodynamic parameters [27]. When ΔH > 0 and ΔS > 0, hydrophobic interactions are the main force. When ΔH < 0 and ΔS < 0, the van der Waals force and hydrogen bonds are the major non-covalent forces. When ΔH < 0 and ΔS > 0, an electrostatic force is dominant. Accordingly, the interactions between β-LG and proanthocyanidins A1, A2, and B2 were dominated by van der Waals forces and hydrogen bonds, whereas the main non-covalent force between β-LG and proanthocyanidins B1, B3, and C1 was hydrophobic interaction (Table 1). The dominant force between α-LA and A1, B1, B2, and B3 was similar to that between β-LG and A1, B1, B2, and B3. The interaction between proanthocyanidin A2 and α-LA mainly occurred through hydrophobic bonds. The formation of the α-LA-C1 complex was mainly driven by hydrogen bonds and van der Waals forces (Table 2).

### 2.2. Fourier-Transform Infrared Spectroscopy Analysis

**Figure 4:**
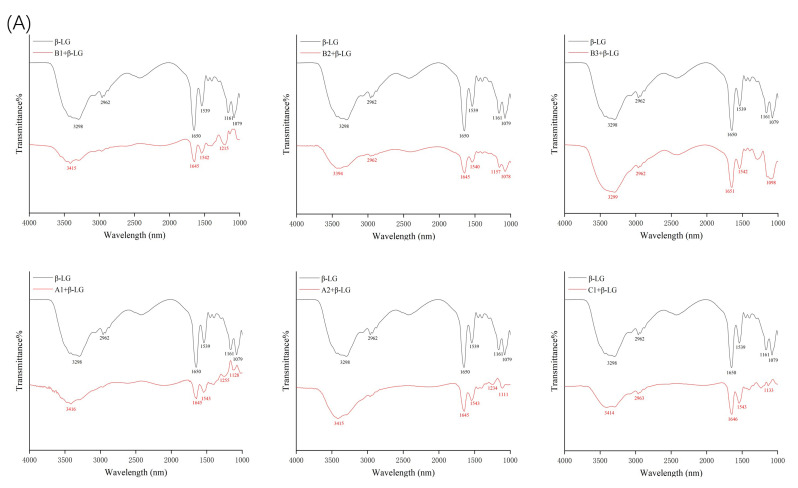

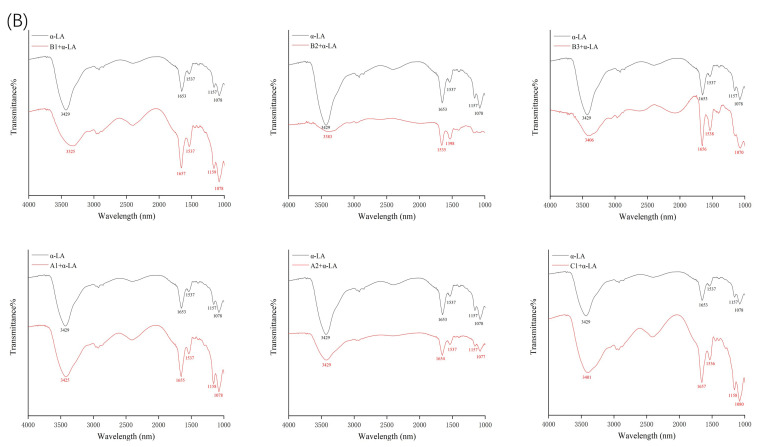
FTIR spectroscopy of β-LG (**A**) or α-LA (**B**) in the presence and absence of proanthocyanidins A1, A2, B1, B2, B3, and C1.

**Figure 5:**
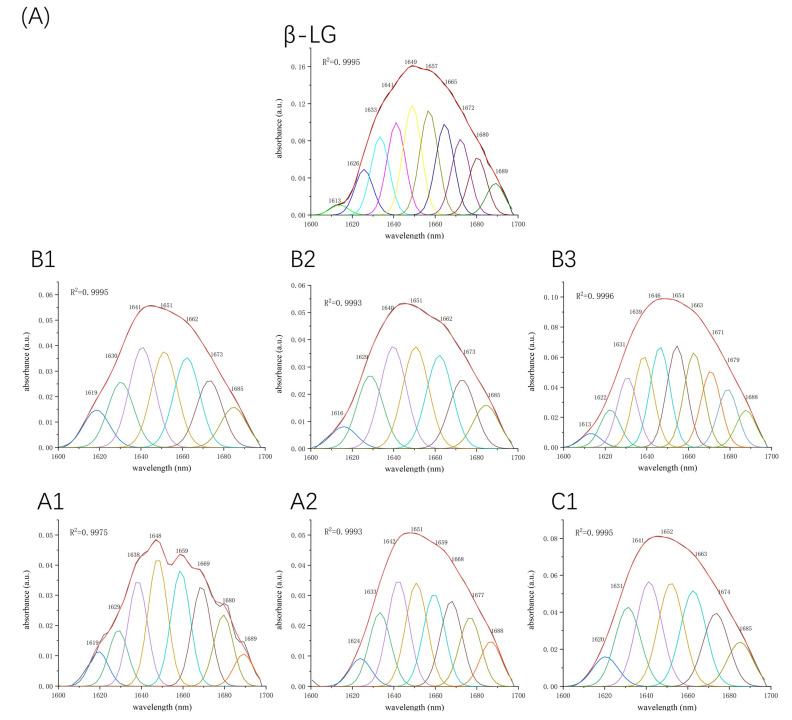

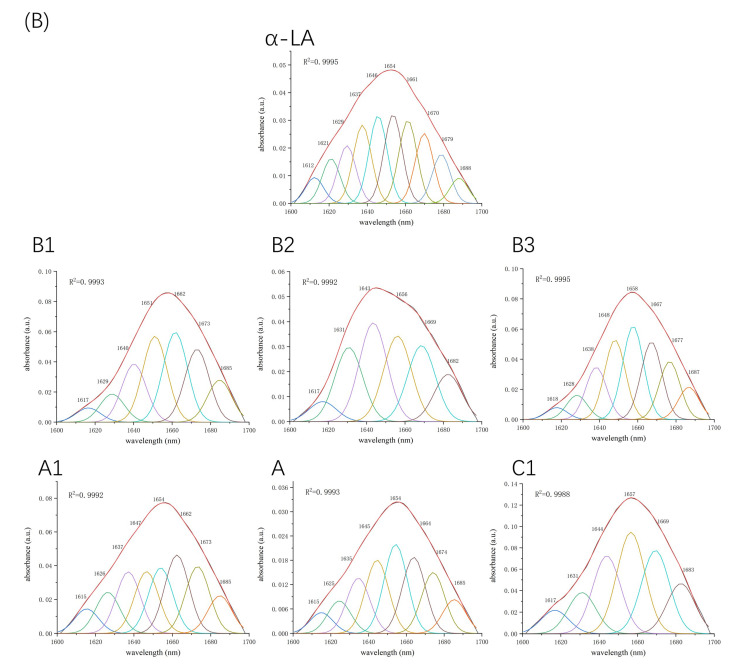
Second derivative analysis and curve-fitted amide I region (1700–1600 cm^−1^) of FTIR spectroscopy for β-LG (**A**) or α-LA (**B**) with or without proanthocyanidins A1, A2, B1, B2, B3, and C1.

Fourier-transform infrared spectroscopy (FTIR) was used to determine the changes in the secondary structure of proteins caused by the formation of the protein–procyanidin complex. The protein FTIR spectra exhibit amide bands that represent vibrations in peptide moieties. Both spectral shape variations and shifting for amide I band (1700–1600 cm^−1^, C=O peptide bond) and the amide II band (1600–1500 cm^−1^, N-H bending and C-N stretching) [28,29] are extremely useful for the identification of secondary structural changes of the protein. As shown in Figure 4A, β-LG shows two major peaks in the 1700–1500 cm^−1^ regions, with amide I at 1650 cm^−1^ and amide II at 1539 cm^−1^. The amide I of β-LG shifted to 1645 cm^−1^ after combining with A1, A2, B1, and B2; to 1646 cm^−1^ after combing with C1; and to 1651 cm^−1^ after combining with B3. Moreover, amide II shifted to 1543 cm^−1^, 1543 cm^−1^, 1542 cm^−1^, 1540 cm^−1^, 1542 cm^−1^, and 1543 cm^−1^, respectively, after combining with A1, A2, B1, B2, B3, and C1. These changes disturbed the secondary structure of β-LG due to binding with proanthocyanidins. In the case of α-LA, even though no obvious change was observed in the position of the peaks after the addition of proanthocyanidins, the alterations in the shape of the amide I and amide II bands were observed (Figure 4B), indicating the changes in the protein secondary structure. To determine the detailed information regarding the changes in the secondary structure of two proteins, Peakfit software was used to separate overlapping peaks and perform fitting calculations [30]. The components of the amide I band were assigned as reported [31]: β-sheet (1613–1637 cm^−1^, 1682–1689 cm^−1)^; random coils (1637–1645 cm^−1^); α-helix (1645–1662 cm^−1^); and β-turn (1662–1682 cm^−1^) (Figure 5). The relative percentage of secondary structural elements in the absence and presence of proanthocyanidins was calculated (Table 3). The data suggest that proanthocyanidins induced more pronounced conformational changes in α-LA than β-LG, which is consistent with the binding affinity obtained from fluorescence spectral analysis.

### 2.3. Circular Dichroism Spectroscopy Analysis

**Figure 6:**
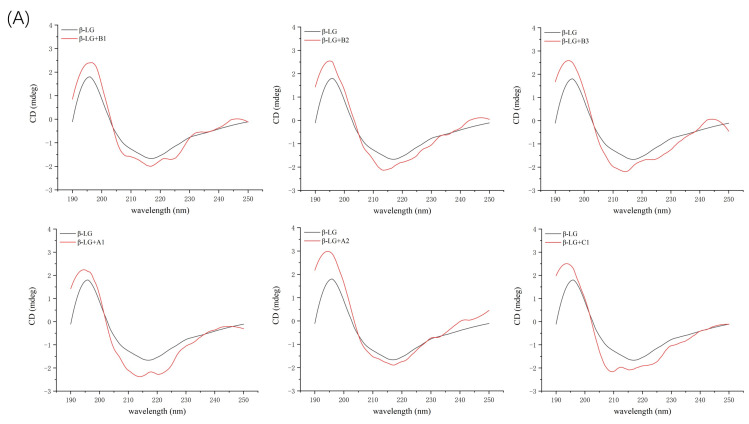

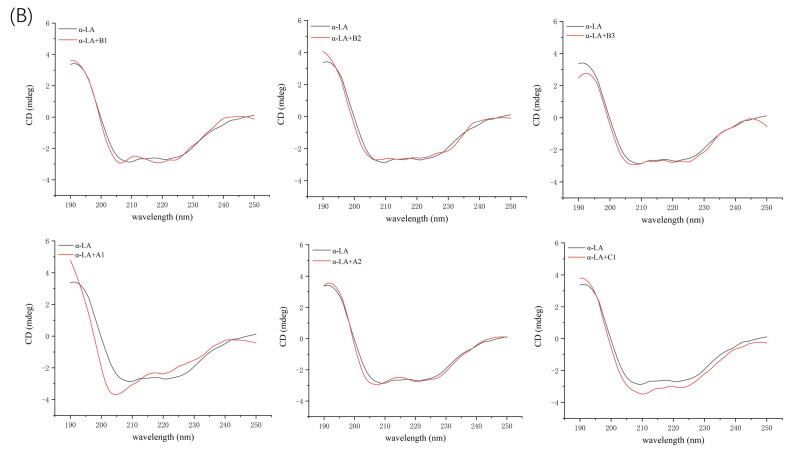
Far ultraviolet CD spectra of β-LG (**A**) or α-LA (**B**) (10 µM) with proanthocyanidin A1, A2, B1, B2, B3, and C1 (50 µM).

Additional evidence for the impact of proanthocyanidin A1, A2, B1, B2, B3, and C1 on the secondary structure of β-LG and α-LA was obtained by circular dichroism (CD) spectra. At pH 6.3 and room temperature, β-LG showed characteristics of β-sheet, as a strong positive band between 195–200 nm and negative broadband in the region of 210–220 nm (Figure 6A). The CD spectrum of α-LA consisted of an α-helix band with double negative peaks at 208 nm and 222 nm and a negative β-sheet broadband around 215 nm (Figure 6B). Similar features of CD spectra of β-LG and α-LA have been previously reported [16,32]. Proanthocyanidins induced changes in band shape and intensity in the far-UV wavelength region, confirming the alteration in the secondary structure of β-LG and α-LA. β-LG showed a decrease in band intensity near 210 nm upon the addition of proanthocyanidin A1, B2, and B3 because of the reduction in β-sheet content and an increase in α-helix content [33]. In the presence of A2, the band shape was changed due to the considerable reduction in β-turn content and increase in α-helix content. The changes in secondary structure induced by proanthocyanidin B1 and C1 were not significant (Table 4), which is consistent with their low K_a_ value of β-LG obtained by fluorescence spectral analysis. In the case of α-LA, the addition of proanthocyanidin A1 considerably induced the lower proportion of α-helix and β-turn, and a higher proportion of β-sheet, leading to an increase in the intensity of the negative broadband near 205 nm. The data verified the high binding affinity of proanthocyanidin A1 as determined by fluorescence spectra analysis. Proanthocyanidin A2 and B2 significantly reduced β-turn content. The significant effect of A2 and B2 on the secondary structure of α-LA seemed to be inconsistent with its low K_a_ value obtained by fluorescence spectra. A similar phenomenon was observed in our previous study, which reported a considerable effect of C1 and A1 on β-casein structures with relatively weak binding affinities [22]. The molecular flexibility and molecule size of oligomeric proanthocyanidins were also considerable factors affecting the secondary structure of proteins in protein–proanthocyanidin complexes. The effects of other proanthocyanidins on the structure of α-LA were not significant.

In general, all six proanthocyanidins rearranged the pattern of secondary structures. The extent and pattern of secondary structure transitions obtained by CD spectra supported the results obtained by FTIR analysis. Similar changes in structures of whey protein induced by other polyphenols have been previously reported. For example, Al-Hanish et al. [16] showed that the formation of an EGCG–protein complex caused the transition of α-LA from α-helix to β-sheet. Kanakis et al. [34] compared the effects of different tea polyphenols on β-LG and found larger perturbations of protein secondary structure induced by larger and bulkier polyphenols. These changes in the secondary structure of whey proteins were caused by the binding of polyphenols to the amino acid residues of proteins via hydrogen bonds and hydrophobic interactions, which resulted in a loss of the initial hydrogen network structure of proteins and rearrangement [9].

### 2.4. Molecular Docking Analysis

**Figure 7:**
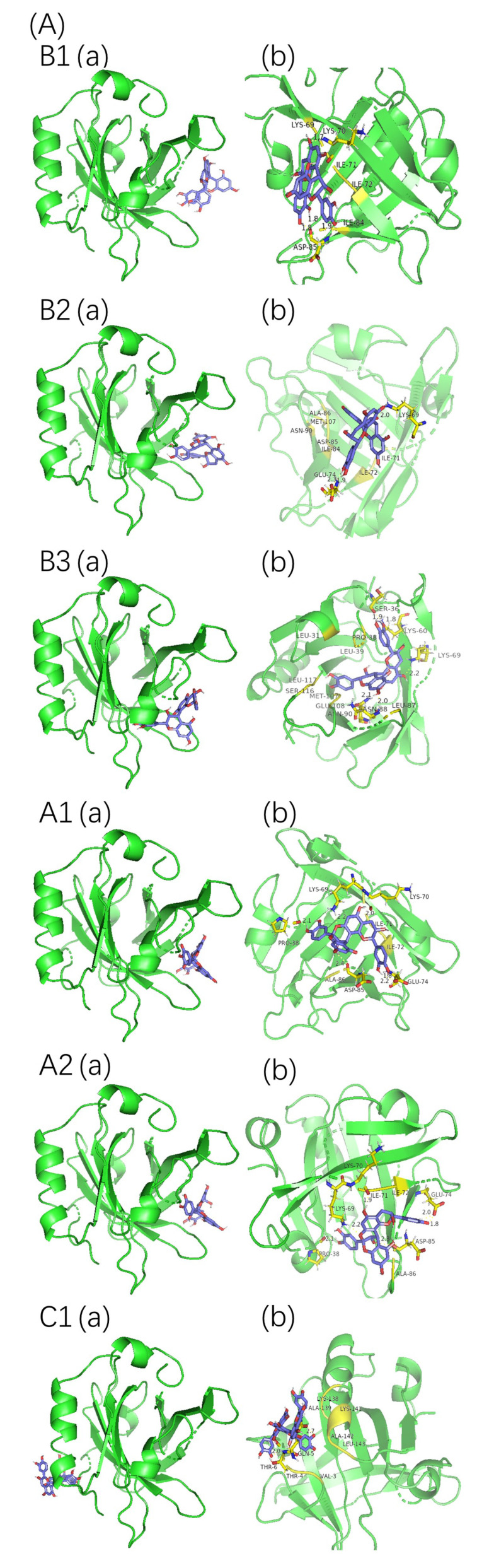

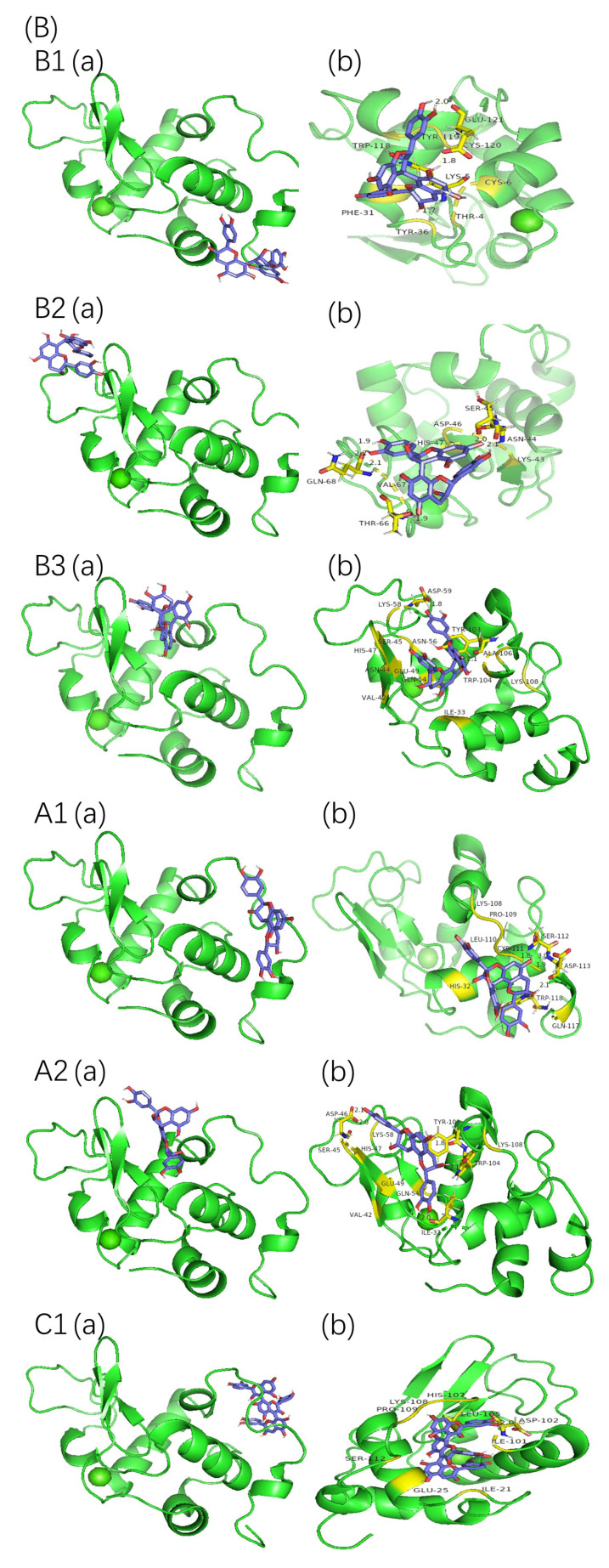
Molecular docking results of proanthocyanidins A1, A2, B1, B2, B3, and C1 with β-LG (**A**) or α-LA (**B**). (**a**) Best conformation for proanthocyanidins with proteins; (**b**) binding sites in detail. The interaction of amino acid residues with proanthocyanidins are shown in yellow.

Protein–proanthocyanidin complexes were simulated and molecular docking was performed to identify the possible binding sites between β-LG or α-LA and oligomeric proanthocyanidins. The optimum docking results with the lowest energy and highest binding were selected (Figure 7). The parameters for molecular interactions and the binding energies are listed in Table 5 and Table 6.

The best binding site of β-LG for proanthocyanidin A1, A2, B1, B2, and B3 was located at the peripheries of the β-barrel, whereas that for C1 was close to the hydrophobic surface pocket formed between β-sheet and α-helix. The highest fluorescence intensity of β-LG was attributed to Trp19, which is located far from the active site [15]. Thus, the direct interactions between proanthocyanidins and Trp19 of β-LG were not observed during molecular docking, and they were consistent with the light red shift observed in the fluorescence spectra. In the case of α-LA, the high-affinity binding sites were located on the side of α-helical domains (for A1, B1, and C1), the side of β-sheet domains (for B2), and the cleft between α-helical and β-sheet domains (for A2 and B3). All six types of proanthocyanidins were in the vicinity with and interacted with Trp 104/Trp 118/Trp 60 of α-LA, and these interactions may explain the offset of λ_max_ observed in the fluorescence spectra [35]. The binding sites of β-LG or α-LA for proanthocyanidins derived from molecular docking are consistent with the potential active sites reported in previous studies [16,36,37].

The non-covalent interactions between β-LG/α-LA and A1, A2, B1, B2, B3, and C1 were mainly hydrophobic forces, hydrogen bonds, and van der Waals forces. The negative ΔG values show that the interactions between the proteins and proanthocyanidins were spontaneous at room temperature. The ΔG values for interactions between β-LG and proanthocyanidins obtained by molecular docking and fluorescence spectra analysis were in the following order: B1 > C1 > B2 > B3 > A2 > A1. The ΔG values for interactions between α-LA and proanthocyanidins were in the following order: C1 > B2 > B1 > A2 > B3 > A1. These orders differed from those of K_a_ values obtained by fluorescence spectroscopy. The condition in which proteins were set as rigid molecules without solvent during docking was distinct from the actual experiment conducted with phosphate buffer solvent [38]. In particular, α-LA has a higher random coil ratio and less rigidity, which may contribute to the difference in the results between molecular docking and fluorescence spectra analysis.

### 2.5. Effects of WPI on the Stability of Proanthocyanidins during In Vitro Gastrointestinal Digestion

**Figure 8:**
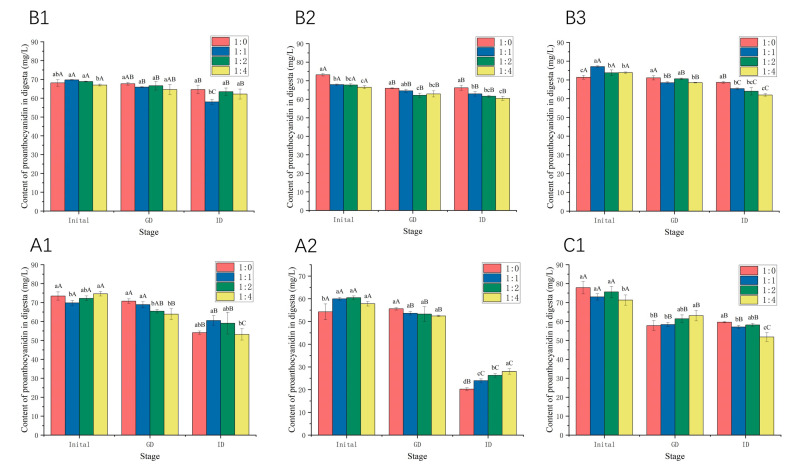
The stability of proanthocyanidin A1, A2, B1, B2, B3, and C1 with WPI at molar ratios of 1:0, 1:1, 1:2, and 1:4. GD: gastric-digested; ID: gastrointestinal-digested. Different lowercase letters indicate that there are significant differences in the same stage of samples at different molar ratios, *p* < 0.05. Different uppercase letters indicate that there are significant differences in the different stage of samples at the same molar ratio, *p* < 0.05.

The changes in the content of proanthocyanidins during gastrointestinal digestion in the absence and presence of WPI were studied. As shown in Figure 8, the content of proanthocyanidins decreased after in vitro digestion, and the final retention rates of proanthocyanidins A1, A2, B1, B2, B3, and C1 were 73.67%, 37.27%, 94.82%, 90.40%, 96.18%, and 76.60%, respectively. Generally, proanthocyanidins are stable in an acidic environment, and degradation occurs in the mildly alkaline intestinal environment [39]. The stability of A-type and C-type proanthocyanidins was much poorer than that of B-type during the process of gastrointestinal digestion, especially A2 (the content of proanthocyanidin A2 decreased from 54.26 mg/L to 20.22 mg/L). The results are consistent with those of our previous study [22]. A1 is composed of (−)-epicatechin and (+)-catechin units, whereas A2 is composed of two (−)-epicatechin units, and the joint style is common. In an alkaline environment, the stability of (−)-epicatechin is lower than (+)-catechin [40], which may cause the different stabilities of A1 and A2 in the process of gastrointestinal digestion. The addition of WPI increased the content by 11.90% for A1 and by 38.43% for A2, which is much higher than for other proanthocyanidins (Figure 8). The stability of the proanthocyanidins tested during the process of gastrointestinal digestion was consistent with their binding constant K_a_ with β-LG/α-LA, except for A2 (Table 1 and Table 2). Though the K_a_ of α-LA-A2 was relatively lower, proanthocyanidin A2 induced significant changes in the secondary structures of α-LA, indicating the interaction between α-LA and A2, which contributes to the protective effects on A2. The WPI–proanthocyanidin complexes limited the release of proanthocyanidins during gastric digestion, leading to potential oxidation in the small intestine. The subsequent digestion of protein in the intestine environment may disrupt these interactions, resulting in an increase in absolute bioaccessibility [41]. In addition, the antioxidant properties of hydrolysates from WPI also protected against the degradation of proanthocyanidins [42]. Similar protective effects were observed in other studies. For example, the addition of a green tea extract to dairy matrices promoted polyphenol–protein complex formation, which significantly improved polyphenol stability in a simulated gastrointestinal environment and enhanced the antioxidant activity [43]. WPI enhanced both the stability and antioxidant activity of blueberry anthocyanins [9]. Moreover, the tryptic digest of α-LA interacted with berry procyanidins and prevented the degradation of procyanidins [12]. Our previous research also found that milk casein increased the retention rates of procyanidins B1 and B2 [22]. In contrast, the addition of WPI reduced the stability of B2 and B3 and did not significantly affect that of B1 and C1. Some previous studies also demonstrated the negative effect of milk proteins on the stability of tea flavan-3-ols [41,44,45], as well as phenolic acids in coffee [46]. The antioxidant activity of polyphenols also decreased in the presence of milk protein [25,47]. These conflicting findings may be associated with types and conformations of polyphenols and proteins. Overall, WPI may have the potential to protect A-type proanthocyanidins, in particular, proanthocyanidin A2, during gastrointestinal digestion.

### 2.6. Effect of WPI on the Stability of Proanthocyanidins during Storage at Room Temperature

**Figure 9:**
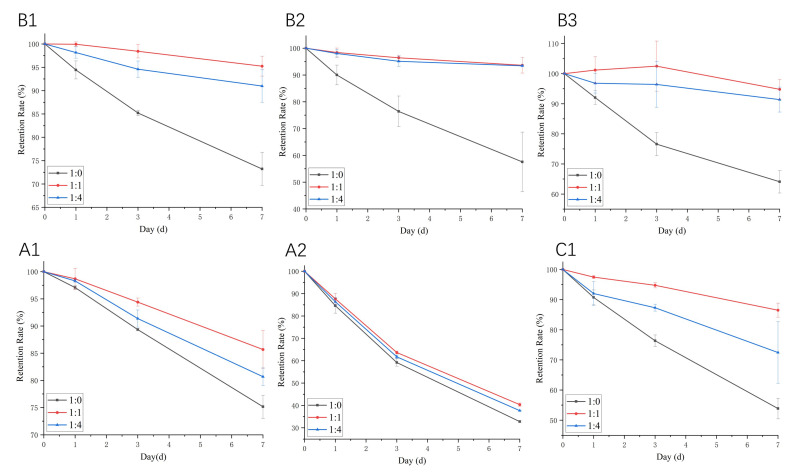
The storage stability of proanthocyanidin A1, A2, B1, B2, B3, and C1 with WPI at room temperature. The molar ratios of proanthocyanidin:WP are 1:0, 1:1, and 1:4, respectively.

During storage, exposure to room temperature may lead to a gradual degradation of proanthocyanidins. Therefore, we examined the stability of the WPI–proanthocyanidin complex during seven-day storage at room temperature. The mixtures were shielded from light to simulate practical storage conditions. As shown in Figure 9, the content of the tested proanthocyanidins in samples without WPI decreased significantly with the extension of storage time. Among the six proanthocyanidins, A2 was the most unstable one during storage, and the retention rate at day 7 was as low as 32.76%. The addition of WPI (molar ratio of 1:1) increased the retention rate of proanthocyanidins A1, A2, B1, B2, B3, and C1 by 14.01%, 23.14%, 30.09%, 62.67%, 47.92%, and 60.56%, respectively. However, the degree of the protection effect of WPI on these six proanthocyanidins was not consistent with the order of K_a_ values of whey proteins with the six proanthocyanidins. This protection was related to the alteration of the secondary structures of the two main components of whey protein (Table 4). WPI protects proanthocyanidins in its protein cavity, preventing degradation [9]. Many studies suggested that the interaction with proteins is an effective method for improving the stability of polyphenols. He et al. [48] found that whey proteins significantly prevented the color loss and degradation of anthocyanins from heat treatment, oxidation, and illumination, which are associated with the binding interaction between anthocyanins and whey proteins. Chung et al. [49] demonstrated that the stability of anthocyanin in model beverages, stored under accelerated storage conditions, could be prolonged with the addition of WPI. Liang et al. [50] emphasized the thermal and acid stability of resveratrol in the presence of β-LG. However, the higher content of WPI had a relatively weak protective effect (Figure 9). This could be explained by the masking effect of WPI on proanthocyanidins, which made proanthocyanidins undetectable using HPLC analysis [51].

## 3. Materials and Methods

### 3.1. Materials

Proanthocyanidins B1, B2, B3, A1, A2, and C1 were purchased from Chengdu Caoyuankang Bio-technology Co., Ltd. (Chengdu, China). Whey protein isolate (WPI, purity ≥ 80%) was purchased from Shanghai Macklin Biochemical Co., Ltd. (Shanghai, China). β-LG and α-LA from bovine milk (purity ≥ 90% and ≥85%, respectively), pepsin from porcine gastric mucosa (≥400 units/mg protein), and pancreatin from porcine pancreas (4 × United States Pharmacopeia specifications) were purchased from Sigma-Aldrich Chemical Co. (St. Louis, MO, USA). Bile salt was purchased from Shanghai Shengong Biological Technology Co., Ltd. (Shanghai, China). Other analytical-grade reagents were purchased from SinoPharm CNCM Ltd. (Shanghai, China).

### 3.2. Fluorescence Spectroscopy

The fluorescence spectra of the samples were obtained using a steady-state and time-resolved fluorescence spectrofluorometer (QM/TM/IM, Photon Technology International, Birmingham, NJ, USA), equipped with a quartz cuvette with an optical path length of 1 cm. Samples were prepared by blending the stock solutions of WPI, β-LG, and α-LA and proanthocyanidins (B1, B2, B3, A1, A2, and C1) dissolved in phosphate-buffered saline (PBS) solution (10 mM, pH 6.3). The final concentration of WPI was 20 μM, and it was combined with proanthocyanin concentrations of 0, 10, 20, 40, and 80 μM. The final concentration of β-LG/α-LA was 10 μM, and it was combined with 0, 10, 20, 30, 40, and 50 μM proanthocyanins. The recorded emission signals of the samples ranged from 300 nm to 500 nm at 297 K, 304 K, and 311 K, respectively, and the excitation wavelength was 280 nm. Excitation and emission slit widths were 5 nm. The blank spectrum of the corresponding concentration of proanthocyanidin solution was subtracted.

Fluorescence quenching was calculated using the Stern–Volmer equation [52]:

(1) $$\frac{F_{0}}{F}=1+{\;K}_{\mathrm{sv}}\times \left[Q\right]=1+{\;K}_{q}\times {\;\tau }_{0\;}\times \left[Q\right]$$

where F_0_ and F are the fluorescence intensities of the proteins in the absence or presence of proanthocyanidins (quencher), respectively; K_sv_ is the Stern–Volmer quenching constant; [Q] is the concentration of quencher; K_q_ is the bimolecular quenching constant; and τ_0_ is the lifetime of the fluorophore when the quencher is absent, and the value typically equals to 10^−8^ s.

For static quenching, the value of the association constant (K_a_) and the number of binding sites (n) were calculated by using the modified Stern–Volmer equation given below in the logarithmic form [53]:

(2) $$\log\;[(F_{0}\;-\;F)/F]\;={\;\mathrm{logK}}_{a}\;+\;\mathrm{nlog}\left[Q\right]$$

Thermodynamic parameters were calculated using the van’t Hoff equation [54] as follows:

(3) $${\mathrm{lnK}}_{a}\;=\;-\frac{\Delta H}{\mathrm{RT}}\;+\;\frac{\Delta S}{R}$$

(4) ΔG = ΔH − TΔS

where ΔH is the enthalpy change, ΔG is the Gibbs free energy change, ΔS is the entropy change, R is the gas constant (8.314 J mol^−1^·K^−1^), and T is the absolute temperature.

### 3.3. Fourier-Transform Infrared Spectroscopy

FTIR of the samples was performed using a Nicolet 6700 IR infrared spectrometer (Thermo Scientific, Waltham, MA, USA). The protein–proanthocyanidin mixture (molar ratio of 1:5), individual proanthocyanidin (B1, B2, B3, A1, A2, and C1), and protein (β-LG and α-LA) solutions were prepared in PBS (10 mM, pH 6.3) and then freeze-dried for 30 h by Vacuum Freeze Dryer-2000 (Bilon, Shanghai, China). The freeze-dried powder was blended with KBr at a mass ratio of 3:100 and pressed to form tablets for further measurement. The spectra were recorded in absorbance mode between 400 and 4000 cm^−1^ at a resolution of 4 cm^−1^, and 16 scans were performed using the Nicolet Omnic v8.0 software (Thermo Nicolet Corporation, Waltham, MA, USA). The baselines were corrected automatically. The infrared difference spectra were recorded by subtracting the spectra of the PBS solution from those of the protein solution or by subtracting the spectra of the proanthocyanidin solution from those of the protein–proanthocyanidin mixture solution.

To determine the effects of proanthocyanidins on the secondary structure of two proteins, the spectral region between 1600 cm^−1^ and 1700 cm^−1^ was selected. The FTIR spectra were smoothed with 13 points, and second derivative calculations were performed to acquire the relative percentage of secondary structural elements using the PeakFit Version 4.12 software (SPSS Inc., Chicago, IL, USA) [55].

### 3.4. Circular Dichroism Spectroscopy

CD spectroscopy was performed using the J-1500 spectropolarimeter (Tokyo, Japan) with a constant nitrogen flush at 298 K. The concentration of β-LG or α-LA was 2 μM in PBS (2 mM, pH 6.3) in the absence or presence of proanthocyanidin (10 µM). The spectra of PBS or proanthocyanidins were subtracted as baseline. Spectra were measured in the far-UV region (190–250 nm), with the quartz cell having an optical path length of 1 mm. Each recorded spectrum was an average of three consecutive scans, logging at the scan rate of 50 nm/min. The spectral resolution was 0.2 nm; the response time was 0.25 s; and the slit width was 1 nm. The secondary structure of proteins was estimated by the CDNN program (accessed on 10 November 2020, http://gerald-boehm.de/download/cdnn) [56].

### 3.5. Molecular Docking

Molecular docking calculations were performed using AutoDock 4.2 and its visual docking assistant software AutoDock Tools 1.5.6 (accessed on 19 July 2020, http://autodock.scripps.edu/). The three-dimensional structural data of β-LG (PDB ID: 3NPO) [56] and α-LA (PDB ID: 1HFX) [57] were obtained from the Protein Data Bank (accessed on 23 July 2020, http://www.rcsb.org). The structural parameters of proanthocyanidins (B1, B2, B3, A1, A2, and C1) were downloaded from the PubChem database (accessed on 23 July 2020, https://pubchem.ncbi.nlm.nih.gov/) (PubChem CIDs as 11250133, 122738, 146798, 9872976, 124025, and 169853, respectively). All of the water molecules were removed, polar hydrogen atoms were added, and the charge was adjusted. All of the rotatable torsions for proanthocyanidins were activated, whereas the proteins were assumed to be rigid. To recognize all of the potential binding sites of proteins, grid point networks with a grid interval of 0.375 Å were set to β-LG (X, Y, Z: 120, 120, 120) and α-LA (X, Y, Z: 100, 100, 120). Based on the Lamarckian genetic algorithm, 50 runs were performed for the ligands with 150 individuals in the population; the maximum number of energy evaluations and generations was 2.5 × 10^7^. Other parameters were set to the default values [58]. According to the principles of the lowest binding energy, the optimal docking result was obtained and further analyzed using PyMol 2.4 (accessed on 13 October 2020, https://pymol.org).

### 3.6. Assessment of Stability of Proanthocyanidin in WPI–Proanthocyanidin Mixture during In Vitro Gastrointestinal Digestion

In vitro digestion was carried out according to Wootton-Beard et al. [59]. The proanthocyanidin solutions (0.2 mg/mL) were mixed with the WPI solution in PBS (0.01 M, pH 6.3) at molar ratios of 1:0, 1:1, 1:2, and 1:4, respectively, to create a final volume of 5 mL. Then, the solution was adjusted to pH 2.0 with 1 M HCl, and 1 mL of pepsin (>16,000 U, dissolved in 0.1 M HCl) was added. The mixtures were flushed with nitrogen and incubated at 37 °C in a shaking water bath for 1 h in the dark. The digestion solution was adjusted to pH 5.3 using 0.9 M NaHCO_3_ to end the gastric digestion. The pH was further adjusted to 7.2 with 0.1 M NaOH. Two milliliters of trypsin (5 mg/mL, dissolved in 0.1 M NaHCO_3_) and 2 mL of bile salt solution (25 mg/mL, dissolved in 0.1 M NaHCO_3_) were added. Then, the mixtures were flushed with nitrogen and incubated at 37 °C in a shaking water bath for 2 h in the dark. During digestion, aliquots of WPI–proanthocyanidin digest were collected at the initial stage, the end of gastric digestion, and the end of gastrointestinal digestion. The aliquots were submerged in a boiling water bath for 10 min to inactivate the pepsin and pancreatin. Then, the digestion solutions were centrifuged at 12,000× *g* at 4 °C for 15 min, and the supernatant was collected and stored at −80 °C. The stability of proanthocyanidins in the process of gastrointestinal digestion was evaluated by HPLC, and the stability was defined as the content of proanthocyanidins (mg/L) recovered in the final digest [41].

### 3.7. Assessment of the Storage Stability of Proanthocyanidins in WPI–Proanthocyanidin Mixture

The proanthocyanidin solutions (0.2 mg/mL) were mixed with the WPI solution in PBS (0.01 M, pH 6.3) at molar ratios of 1:0, 1:1, and 1:4, respectively. The mixture was purged with nitrogen and stored in the dark at room temperature for 7 days. Aliquots (1.5 mL) of solutions from each group on day 0, 1, 3, and 7 were collected, and the proanthocyanidin content was analyzed using HPLC. The storage stability of proanthocyanidins was evaluated via their retention rate (%), which is the ratio of residual proanthocyanidin content after storage versus the initial proanthocyanidin content.

### 3.8. High-Performance Liquid Chromatography (HPLC) Analysis of Proanthocyanidins

According to the method from our previous study with appropriate modifications [22], the conditions were as follows: The samples were separated on a C18 column (5 μm, 250 × 4.6 mm) at a temperature of 25 °C and a pressure of 10.2 MPa. The eluents were composed of phosphoric acid/water (solvent A) (1/1000, *v/v*) and acetonitrile (solvent B). The mobile phase gradient was as follows: 0–25 min, linear gradient from 90% A to 75% A; 25–28 min, isocratic 90% A. The flow rate was 1 mL/min. Proanthocyanidin B1, B2, B3, A1, A2, and C1 were detected at 280 nm using a Waters Acquity PDA detector (Waters Corporation, Manchester, UK). The chromatograms were integrated by employing EMPOVER software (Waters Corporation, Manchester, UK). The standard curve lines of pure proanthocyanidins were used to quantify the peaks of the digested samples.

### 3.9. Statistical Analysis

All of the experiments were performed in triplicate. Data are expressed as the mean ± standard deviation. The mean values were compared using one-way ANOVA and the least significant difference (LSD) tests, which were performed using SPSS 26. A *p*-value of <0.05 was considered to be statistically significant. All of the figures were prepared using Origin 2019.

## 4. Conclusions

In this study, the interactions between oligomeric proanthocyanidins, such as B1, B2, B3, A1, A2, and C1, and two whey proteins (β-LG and α-LA) were explored by multi-spectroscopic and molecular docking methods. Fluorescence spectroscopy analysis showed that proanthocyanidins formed a complex with whey proteins. At 297 K, the K_a_ values were arranged as A1 > A2 > C1 > B2 > B1 > B3 for β-LG, whereas those for α-LA were arranged as A1 > B1 > C1 > B2 > B3 > A2. The K_a_ value of α-LA with proanthocyanidins was higher than that of β-LG with proanthocyanidins (except for A2). The main non-covalent forces between β-LG/α-LA and six proanthocyanidins were hydrophobic interactions or van der Waals forces and hydrogen bonds. FTIR and CD analyses showed that proanthocyanidins rearranged the pattern of secondary structures of β-LG and α-LA. The effect of proanthocyanidins on the α-LA structure was more prominent (except for A2) than on the β-LG structure. WPI has the potential to protect A-type proanthocyanidins, particularly proanthocyanidin A2, during gastrointestinal digestion. The degradation of proanthocyanidins during storage at room temperature was significantly inhibited by WPI. This study highlights the potential use of polyphenol–protein complexes as a functional food supplement or carrier for targeted delivery in the body.

## Figures and Tables

**Table 1:** Quenching constants, binding constants, and thermodynamic parameters for proanthocyanidin A1, A2, B1, B2, B3, and C1 binding to β-LG at 297, 304, and 311 K.

	T/K	Ksv (×10^4^ L mol^−1^)	Kq (×10^12^ L mol^−1^ s^−1^)	n	Ka (×10^3^ L mol^−1^)	∆H (KJ mol^−1^)	∆S (KJ mol^−1^ K^−1^)	∆G (KJ mol^−1^)
A1	297	1.61 ± 0.08 ^aB^	1.61 ± 0.08 ^aB^	1.06 ± 0.04	29.51 ± 4.47 ^aA^	−40.45	−0.05	−25.45
	304	1.59 ± 0.06 ^a^	1.59 ± 0.06 ^a^	1.04 ± 0.03	22.22 ± 1.97 ^a^			−25.09
	311	1.52 ± 0.07 ^a^	1.52 ± 0.07 ^a^	1.00 ± 0.06	14.51 ± 3.27 ^ab^			−24.74
A2	297	1.16 ± 0.02 ^aC^	1.16 ± 0.02 ^aC^	1.04 ± 0.03	18.59 ± 2.41 ^aB^	−134.21	−0.37	−24.40
	304	1.06 ± 0.01 ^b^	1.06 ± 0.01 ^b^	0.95 ± 0.02	6.50 ± 0.53 ^b^			−21.82
	311	1.01 ± 0.03 ^c^	1.01 ± 0.03 ^c^	0.82 ± 0.03	1.61 ± 0.22 ^b^			−19.23
B1	297	0.90 ± 0.01 ^aD^	0.90 ± 0.01 ^aD^	0.95 ± 0.04	5.60 ± 0.75 ^cD^	55.03	0.26	−21.30
	304	0.86 ± 0.02 ^b^	0.86 ± 0.02 ^b^	1.02 ± 0.01	10.81 ± 0.23 ^b^			−23.10
	311	0.85 ± 0.01 ^b^	0.85 ± 0.01 ^b^	1.05 ± 0.03	14.97 ± 0.40 ^a^			−24.90
B2	297	0.88 ± 0.01 ^aD^	0.88 ± 0.01 ^aD^	0.98 ± 0.01	7.54 ± 0.29 ^aCD^	−72.97	−0.17	−22.48
	304	0.85 ± 0.02 ^b^	0.85 ± 0.02 ^b^	0.91 ± 0.05	3.40 ± 0.56 ^b^			−21.29
	311	0.82 ± 0.01 ^c^	0.82 ± 0.01 ^c^	0.87 ± 0.03	2.02 ± 0.24 ^c^			−20.10
B3	297	0.95 ± 0.02 ^aD^	0.95 ± 0.02 ^aD^	0.95 ± 0.04	5.59 ± 0.69 ^bD^	39.53	0.21	−22.84
	304	0.82 ± 0.01 ^b^	0.82 ± 0.01 ^b^	1.01 ± 0.02	9.19 ± 0.50 ^ab^			−24.31
	311	0.80 ± 0.01 ^b^	0.80 ± 0.01 ^b^	1.03 ± 0.02	11.35 ± 0.75 ^a^			−25.78
C1	297	1.93 ± 0.04 ^aA^	1.93 ± 0.04 ^aA^	0.92 ± 0.05	8.30 ± 1.49 ^bCD^	103.77	0.42	−22.27
	304	1.53 ± 0.02 ^b^	1.53 ± 0.02 ^b^	1.03 ± 0.03	22.07 ± 2.08 ^b^			−25.24
	311	1.47 ± 0.03 ^b^	1.47 ± 0.03 ^b^	1.13 ± 0.03	55.44 ± 6.38 ^a^			−28.21

Different lowercase letters indicate that there are significant differences in the same group of proanthocyanidins at different temperatures, *p* < 0.05. Different uppercase letters indicate that there are significant differences in the different groups of proanthocyanidins at 297 K, *p* < 0.05.

**Table 2:** Quenching constants, binding constants, and thermodynamic parameters for proanthocyanidins A1, A2, B1, B2, B3, and C1 binding to α-LA at 297, 304, and 311 K.

	T/K	K_sv_ (×10^4^ L mol^−1^)	K_q_ (×10^12^ L mol^−1^ s^−1^)	n	K_a_ (×10^4^ L mol^−1^)	∆H (KJ mol^−1^)	∆S (KJ mol^−1^ K^−1^)	∆G (KJ mol^−1^)
A1	297	2.14 ± 0.08 ^aC^	2.14 ± 0.08 ^aC^	1.19 ± 0.05	14.72 ± 2.36 ^aA^	−112.12	−0.28	−29.13
	304	1.78 ± 0.02 ^b^	1.78 ± 0.02 ^b^	1.08 ± 0.00	4.01 ± 0.06 ^b^			−27.17
	311	1.68 ± 0.05 ^c^	1.68 ± 0.05 ^c^	1.02 ± 0.04	1.90 ± 0.27 ^b^			−25.22
A2	297	1.61 ± 0.05 ^aD^	1.61 ± 0.05 ^aD^	0.79 ± 0.04	0.19 ± 0.03 ^bD^	41.89	0.20	−18.56
	304	1.58 ± 0.05 ^a^	1.58 ± 0.05 ^a^	0.82 ± 0.04	0.26 ± 0.4 ^b^			−19.97
	311	1.40 ± 0.03 ^b^	1.40 ± 0.03 ^b^	0.88 ± 0.04	0.41 ± 0.06 ^a^			−21.40
B1	297	2.52 ± 0.05 ^aA^	2.52 ± 0.05 ^aA^	1.07 ± 0.04	5.04 ± 0.71 ^bB^	67.90	0.32	−26.67
	304	2.46 ± 0.05 ^a^	2.46 ± 0.05 ^a^	1.13 ± 0.02	9.21 ± 0.63 ^ab^			−28.90
	311	2.15 ± 0.03 ^b^	2.15 ± 0.03 ^b^	1.19 ± 0.09	18.85 ± 5.88 ^a^			−31.13
B2	297	2.11 ± 0.05 ^aC^	2.11 ± 0.05 ^aC^	1.06 ± 0.06	4.13 ± 0.85 ^aBC^	−32.94	−0.02	−26.12
	304	1.77 ± 0.04 ^b^	1.77 ± 0.04 ^b^	1.05 ± 0.04	2.89 ± 0.35 ^ab^			−25.96
	311	1.57 ± 0.03 ^c^	1.57 ± 0.03 ^c^	1.03 ± 0.04	2.20 ± 0.27 ^b^			−25.80
B3	297	1.26 ± 0.03 ^aE^	1.26 ± 0.03 ^aE^	1.01 ± 0.04	1.46 ± 0.20 ^aCD^	13.61	0.13	−23.61
	304	1.22 ± 0.03 ^a^	1.22 ± 0.03 ^a^	1.03 ± 0.03	1.62 ± 0.20 ^a^			−24.49
	311	1.11 ± 0.02 ^b^	1.11 ± 0.02 ^b^	1.05 ± 0.03	1.86 ± 0.21 ^a^			−25.37
C1	297	2.33 ± 0.06 ^aB^	2.33 ± 0.06 ^aB^	1.07 ± 0.05	4.95 ± 0.87 ^aB^	−29.10	−0.01	−26.60
	304	2.09 ± 0.05 ^b^	2.09 ± 0.05 ^b^	1.05 ± 0.04	3.66 ± 0.49 ^a^			−26.54
	311	1.98 ± 0.03 ^b^	1.98 ± 0.03 ^b^	1.04 ± 0.04	2.89 ± 0.45 ^a^			−26.48

Different lowercase letters indicate that there are significant differences in the same group of proanthocyanidins at different temperatures, *p* < 0.05. Different uppercase letters indicate that there are significant differences in the different groups of proanthocyanidins at 297 K, *p* < 0.05.

**Table 3:** Secondary structure analysis for β-LG/α-LA and their complexes with A1, A2, B1, B2, B3, and C1 by second derivative FTIR method.

Samples	α-Helix%	β-Sheet%	β-Turn%	Coil%
β-LG	30.77	23.79	32.18	13.26
A1+β-LG	37.93	19.02	26.60	16.45
A2+β-LG	32.50	24.27	25.62	17.61
B1+β-LG	37.44	28.85	13.53	20.18
B2+β-LG	38.65	27.47	13.64	20.23
B3+β-LG	29.64	23.31	33.68	13.37
C1+β-LG	37.59	28.83	13.79	19.78
α-LA	28.18	25.13	19.50	27.19
A1+α-LA	47.08	23.55	15.31	14.07
A2+α-LA	20.20	32.15	31.04	16.61
B1+α-LA	44.95	21.54	18.66	14.84
B2+α-LA	21.31	35.19	18.92	24.58
B3+α-LA	40.28	15.93	31.61	12.18
C1+α-LA	26.94	30.32	22.10	20.64

**Table 4:** Secondary structure analysis for β-LG/α-LA and its complexes with A1, A2, B1, B2, B3, and C1 by the CD method.

Samples	α-Helix%	β-Sheet%	β-Turn%	Coil%
β-LG	27.00 ± 2.42 ^c^	29.73 ± 6.80 ^a^	18.80 ± 2.75 ^ab^	24.67 ± 3.71 ^ab^
A1+β-LG	31.07 ± 1.85 ^bc^	15.37 ± 4.10 ^bc^	24.83 ± 2.49 ^a^	28.70 ± 0.89 ^a^
A2+β-LG	41.97 ± 2.90 ^a^	30.70 ± 7.63 ^a^	10.97 ± 2.56 ^c^	16.40 ± 3.40 ^c^
B1+β-LG	32.97 ± 1.37 ^bc^	26.60 ± 3.36 ^abc^	18.40 ± 1.89 ^b^	22.03 ± 1.13 ^bc^
B2+β-LG	37.13 ± 0.55 ^ab^	18.37 ± 0.52 ^abc^	21.77 ± 1.53 ^ab^	22.73 ± 0.73 ^ab^
B3+β-LG	36.07 ± 2.48 ^ab^	14.30 ± 5.05 ^c^	24.67 ± 1.35 ^a^	25.00 ± 1.51 ^ab^
C1+β-LG	28.67 ± 1.56 ^c^	27.03 ± 0.49 ^abc^	19.30 ± 0.75 ^ab^	25.03 ± 0.84 ^ab^
α-LA	27.60 ± 1.25 ^A^	23.83 ± 2.77 ^BC^	12.57 ± 0.67 ^A^	36.07 ± 1.44 ^B^
A1+α-LA	16.87 ± 1.24 ^B^	42.00 ± 3.05 ^A^	0.00 ± 0.00 ^C^	41.13 ± 1.87 ^A^
A2+α-LA	28.27 ± 1.50 ^A^	29.43 ± 3.25 ^B^	7.27 ± 2.09 ^B^	35.03 ± 0.92 ^B^
B1+α-LA	25.77 ± 0.79 ^A^	24.40 ± 2.38 ^BC^	13.87 ± 0.87 ^A^	36.00 ± 1.42 ^B^
B2+α-LA	25.83 ± 2.02 ^A^	29.53 ± 4.01 ^B^	8.47 ± 1.34 ^B^	36.20 ± 2.98 ^B^
B3+α-LA	25.63 ± 1.01 ^A^	21.80 ± 1.37 ^BC^	15.23 ± 0.24 ^A^	37.37 ± 0.67 ^AB^
C1+α-LA	17.00 ± 0.40 ^A^	20.17 ± 1.44 ^C^	15.30 ± 1.38 ^A^	37.57 ± 0.26 ^AB^

Different lowercase letters indicate significant differences of β-LG and its complexes in the content of the same secondary structure type, *p* < 0.05. Different uppercase letters indicate significant differences of α-LA and its complexes in the content of the same secondary structure type, *p* < 0.05.

**Table 5:** Molecular docking parameters for proanthocyanidins binding to β-LG.

Complex	Hydrogen Bonds	H-Bond Distance (Å)	Hydrophobic Residues	Other Residues	ΔG (kJ mol^−1^)	Intermolecular Energy (kJ mol^−1^)	Torsional Energy (kJ·mol^−1^)
A1+β-LG	Pro38, Lys69, Lys70, Glu74 *, Asp85	1.8–2.4	Ala86, Ile71, Ile72	-	−28.13	−41.86	13.73
A2+β-LG	Pro38, Lys69, Lys70, Glu74 *, Asp85	1.8–2.3	Ala86, Ile71, Ile72	-	−26.96	−40.73	13.73
B1+β-LG	Lys70, Asp85 ^#^	1.7–1.9	Ile71, Ile72, Ile84	Lys69	−14.06	−30.31	16.25
B2+β-LG	Lys69, Glu74	1.9–2.3	Ile71, Ile72, Ile84, Ala86, Met107	Asp85, Asn90	−19.46	−35.71	16.25
B3+β-LG	Ser36, Lys60, Lys69, Asn88, Asn90	1.8–2.2	Leu31, Pro38, Leu39, Leu87, Met107, Leu117	Glu108, Ser116	−25.70	−41.94	16.25
C1+β-LG	Gln5, Thr6	2.0–2.7	Val3, Ala139, Ala142, Leu143	Thr4, Lys138, Lys141	−15.57	−40.56	25.01

^#^ These residues form three hydrogen bonds. * These residues form two hydrogen bonds.

**Table 6:** Molecular docking parameters for proanthocyanidins binding to α-LA.

Complex	Hydrogen Bonds	H-Bond Distance (Å)	Hydrophobic Residues	Other Residues	ΔG (kJ·mol^−1^)	Intermolecular Energy (kJ·mol^−1^)	Torsional Energy (kJ·mol^−1^)
A1+α-LA	Ser112 *, Asp133 *, Trp118	1.8–3.0	Pro109, Leu110	His32, Lys108, Cys111, Gln117	−24.91	−38.64	13.73
A2+α-LA	Ile33, Asp46 *, Tyr103, Trp104	1.8–2.1	Val42	Ser45, His47, Glu49, Gln54, Lys58, Lys108	−20.89	−34.62	13.73
B1+α-LA	Lys5 *, Glu121 *	1.7–2.0	Phe31, Trp118	Thr4, Cys6, Tyr36, Tyr119, Cys120	−19.51	−35.75	16.25
B2+α-LA	Asn44, Ser45, Thr66, Gln68 ^#^	1.9–2.2	Val67	Lys43, Asp46, His47	−19.17	−35.41	16.25
B3+α-LA	Tyr103, Asp59	1.8–2.1	Ile33, Val42, Trp104, Ala106	Asn44, Ser45, His47, Glu49, Gln54, Asn56, Lys58, Lys108	−20.93	−37.13	16.25
C1+α-LA	Asp102	2.0	Ile21, Ile101, Leu105, Pro109	Glu25, His107, Lys108, Ser112	−15.07	−40.06	25.01

^#^ These residues form three hydrogen bonds. * These residues form two hydrogen bonds.

## Data Availability

In this study there is no data used.

## Supplementary Materials

Figure S1: The structures of the proanthocyanidins of A1, A2, B1, B2, B3, and C1. Figure S2: Stern–Volmer plots of WPI quenched by proanthocyanidins A1, A2, B1, B2, B3, and C1 at 297 K, 304 K, and 311 K. Figure S3: UV-vis absorption spectra of proanthocyanidin B1, B2, B3, A1, A2, C1, β-LG, and α-LA.
