# Supplementary material for: Elucidation of Interaction between Whey Proteins and Proanthocyanidins and Its Protective Effects on Proanthocyanidins during In-Vitro Digestion and Storage
Source: Molecules. 2021 Sep 8;26(18):5468. doi: 10.3390/molecules26185468 (PMC8471322; doi:10.3390/molecules26185468)
Supplement: Supplementary file 1 [file molecules-26-05468-s001.zip › molecules-1356225-supplementary.pdf]

molecules

MDPI

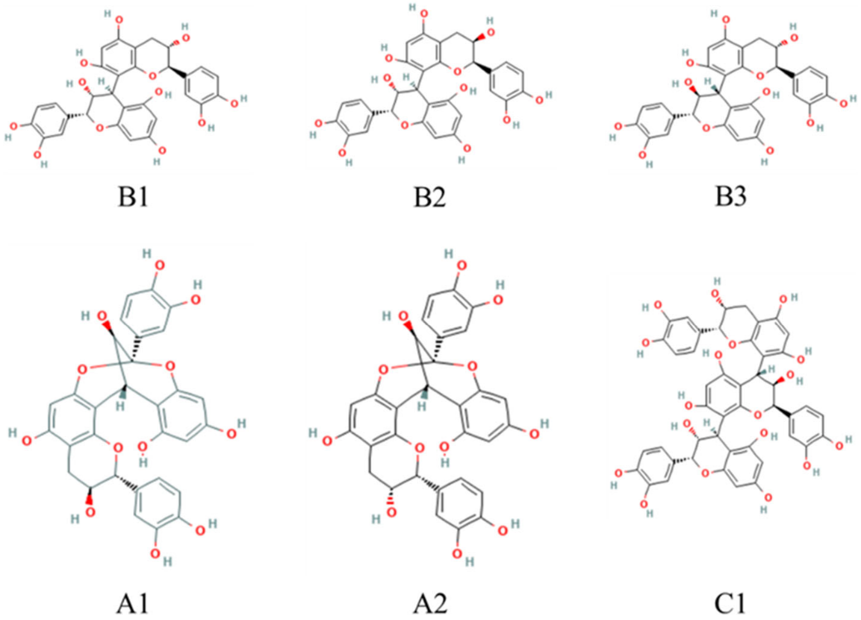

Figure S1. The structures of the proanthocyanidins of A1, A2, B1, B2, B3 and C1.

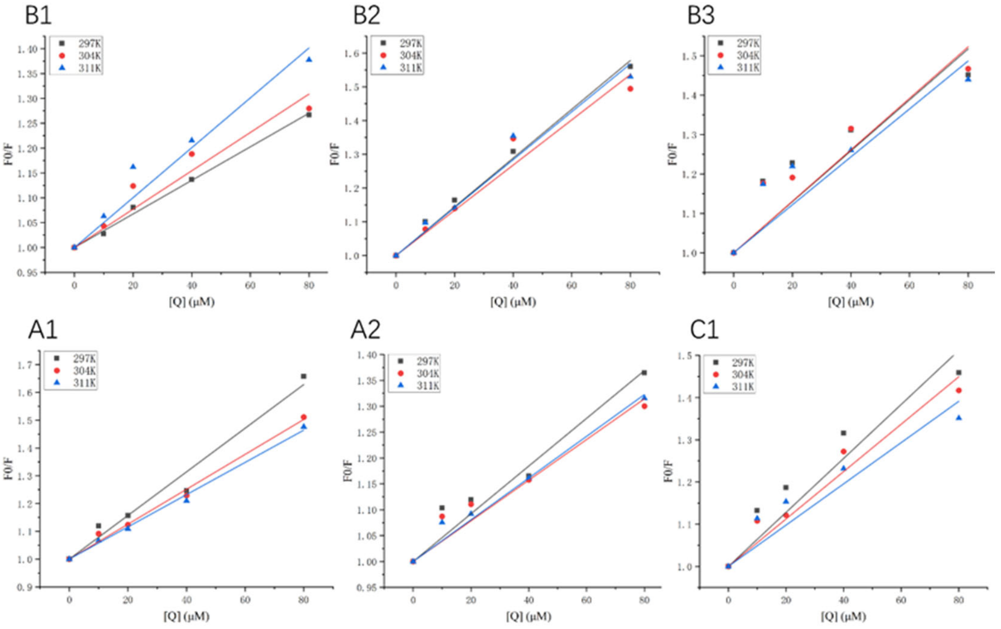

Figure. S2 Stern-Volmer plots of WPI quenched by proanthocyanidins A1, A2, B1, B2, B3 and C1 at 297 K, 304 K, and 311K.

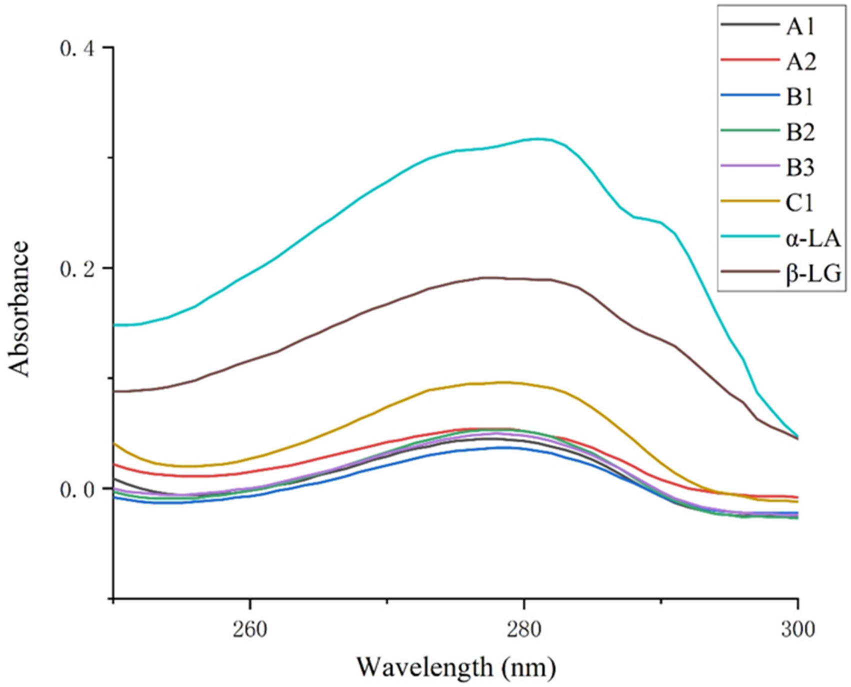

Figure. S3 UV-vis absorption spectra of proanthocyanidin B1, B2, B3, A1, A2, C1, β-LG and α-LA

The UV–vis absorption spectra of proanthocyanidins (B1, B2, B3, A1, A2, and C1), β-LG and α-LA(10 μM) were obtained using a TU-1810 spectrometer (Beijing Purkinje General Co., Ltd., Beijing, China) with the quartz cuvette having the optical path length of 1 cm. PBS (10 mM, pH 6.3) was used as a blank, and the spectra were measured in the range of 250–500 nm at 297 K. The slit width was 1 nm.
